# Notes and News

**Published:** 1892-12-17

**Authors:** 


### NOTES AND NEWS.

COLLECTED AND COMMUNICATED.

ARRANGEMENTS are now being made for the reception of fourteen surgical cases in the Medical School buildings during the closing of the Addenbrooke's Hospital, Cambridge. It has not been found feasible to provide for medical cases, but the out-patients will be treated as usual.

ALTHOUGH the New General Hospital at Birmingham cannot be said to be progressing very fast as far as the structure goes, all arrangements for heating, lighting, and ventilation are being advanced. Mr. Key's system of heating and ventilating as in use at the Victoria Infirmary, Glasgow, is to be adopted. It is estimated that the whole of the appliances for this purpose will require an expenditure of £6,000. The lighting is to be by electricity.

AT the annual dinner of the past and present students of the Dental Hospital of London, Sir Richard Quain occupied the chair, and a distinguished company both medical and dental assembled to do honour to the hospital and the Chairman. The Chairman in his speech said he recognized the importance of the study of teeth for the welfare and comfort and health of mankind, for diseased teeth not only caused local pain, but were fruitful causes of more general disturbances. He pointed out that the mouth was a great breeding house for bacteria and bacilli, which were now proved to be the cause of many disorders, and therefore that a Bacteriological Department was necessary at a dental hospital. In alluding to the history of modern dentistry, he said there were two classes of men who had helped its development, viz., the practical and scientific. Upon this foundation the modern system of dentistry had been established, culminating in the dental hospitals and dental departments of general hospitals, with their schools. Mr. Morton Smale, the Dean of the School, maintained that if the future of the school was to compare favourably with the past, a new hospital must be provided. Such a change would necessitate an expenditure of £40,000, and he had no hesitation in asking the public to help them to provide this sum. Forty donations of £1,000, eighty donations of £500, or one hundred and sixty donations of £250, and the amount is forthcoming. He was willing, and he was sure other gentlemen in the room were also willing to be numbered amongst the one hundred and sixty.

THE Goldsmiths' Company have done more than set an example of generosity by their munificent gift of £25,000 to the Guinness Trust towards the erection of dwellings for the poor. They have carefully selected an excellent object for their charity, judiciously chosen the best means of applying that charity, and robbed their deed of the element of self-glorification. The only important stipulation—namely, that the followers of their craft shall have prior claim—will prove no hampering clause, seeing that many of the poor dwellers in the neighbourhood of Clerkenwell are members of the trade.

THE Dublin Rotunda Hospital, after a justly famous lifetime of a hundred and forty years, is about to have an auxiliary hospital added to it. Not before it is needed, however, for amidst difficulties and disadvantages, owing to inadequate accommodation, has the work been carried on by a devoted staff. This historical institution, one of the first lying-in hospitals in the United Kingdom, has spread its benefits far and near through poor patients seeking shelter within it and by means of the many celebrated men who have added to their experience within its walls. It is therefore to be hoped that the £5,000 appealed for will be rapidly subscribed to render the Rotunda as efficient and worthy of its fame in the future as it has been in the past.

IT may be a source of surprise to many to learn that we advocate the uses of frivolity. But this we especially commend not for the young and giddy—they have it already, and time will do the required toning—but to the scientist, the financier, or the statesman. We argue in fact that without the alleviating power of frivolous moments, the hard-worked brain does not retain the elasticity necessary to the sane mind. By experience we have seen the "clever man" silent, morose, and gloomy who, after living for years on a reputation acquired by the efforts of earlier years of close and unrelaxing study, sink into obscurity and eccentricity. On the other hand we know a veteran whose successful career has been crowned by honours gained for work done after the age of eighty, who takes delight in the worst of puns and jokes, and is eager still over a game of cribbage. Kings have been known, and not the worst of kings either, to indulge in games of leap-frog with their children, and we are inclined to think that a little frivolity in moments of relaxation is as health-giving to the busy mind as a breath of mountain air is to the body.

AT the meeting of The Hospitals Association, on December 7th, Dr. Theodore Ewart, of Colney Hatch, read a paper on the interesting subject of "Epileptic Colonies." Dr. Ewart was able to speak from personal experience of the injurious effect of receiving epileptics into asylums, both on themselves and on the insane patients proper. In advocating the system of colonies for epileptics, which have proved so successful on the Continent, he suggested that the Unions who now pay for the epileptics in asylums should transfer these patients to a colony and pay for them there in a like manner. Dr. Buzzard explained that the National Society for the Employment of Epileptics proposed to establish a colony, but did not anticipate, at any rate, initially to receive the epileptic insane, as they thought it would be prejudicial to the undertaking. An interesting discussion followed, in which Colonel Montefiore, Dr. Ferrier, Mr. Burdett, Dr. Fletcher Beach, and Miss Braunston took part. There were also present at the meeting Lady Frederick Cavendish, Dr. Dawson Williams, Mr. P. Michelli, Mr. Howgrave Graham, and others.

